# Blueprint for a microwave trapped ion quantum computer

**DOI:** 10.1126/sciadv.1601540

**Published:** 2017-02-01

**Authors:** Bjoern Lekitsch, Sebastian Weidt, Austin G. Fowler, Klaus Mølmer, Simon J. Devitt, Christof Wunderlich, Winfried K. Hensinger

**Affiliations:** 1 Department of Physics and Astronomy, University of Sussex, Brighton BN1 9QH, U.K.; 2 Google Inc., Santa Barbara, CA 93117, USA.; 3 Department of Physics and Astronomy, Aarhus University, DK-8000 Aarhus C, Denmark.; 4 Center for Emergent Matter Science, RIKEN, Wako-shi, Saitama 315-0198, Japan.; 5 Department Physik, Naturwissenschaftlich-Technische Fakultät, Universität Siegen, 57068 Siegen, Germany.

**Keywords:** Quantum computing, Quantum Information Processing, quantum technology, ion trapping, surface error correction

## Abstract

The availability of a universal quantum computer may have a fundamental impact on a vast number of research fields and on society as a whole. An increasingly large scientific and industrial community is working toward the realization of such a device. An arbitrarily large quantum computer may best be constructed using a modular approach. We present a blueprint for a trapped ion–based scalable quantum computer module, making it possible to create a scalable quantum computer architecture based on long-wavelength radiation quantum gates. The modules control all operations as stand-alone units, are constructed using silicon microfabrication techniques, and are within reach of current technology. To perform the required quantum computations, the modules make use of long-wavelength radiation–based quantum gate technology. To scale this microwave quantum computer architecture to a large size, we present a fully scalable design that makes use of ion transport between different modules, thereby allowing arbitrarily many modules to be connected to construct a large-scale device. A high error–threshold surface error correction code can be implemented in the proposed architecture to execute fault-tolerant operations. With appropriate adjustments, the proposed modules are also suitable for alternative trapped ion quantum computer architectures, such as schemes using photonic interconnects.

## Teaser

Design to build a trapped ion quantum computer with modules connected by ion transport and voltage-driven quantum gate technology.

## INTRODUCTION

Trapped atomic ions are a very promising candidate for the realization of a universal quantum computer, having demonstrated robust, high-fidelity state preparation (*1*–*3*) and readout (*2*, *4*), high-fidelity universal gate operations (*2*, *5*, *6*), and long qubit coherence times (*2*, *7*).

The concept of using ion transport on microfabricated trap arrays to realize an ion trap quantum computer was proposed by Kielpinski *et al.* (*8*). Developing a comprehensive quantum computing architecture with trapped ions has since attracted a lot of interest. Recently, an approach addressing this was developed by Monroe and Kim (*9*) and Monroe *et al.* (*10*), where ion trap modules, also called elementary logic units, are photonically interconnected using commercial fibers and optical cross-connect switches. This proposal demonstrates that going from one to many modules is within reach of current technology and thereby provides an interesting path to a large-scale ion trap quantum computer. Theoretical investigations of this approach have shown that it can be used for fault-tolerant quantum computing even in the presence of noisy and lossy links (*10*) when combined with entanglement purification (*11*).

An important challenge toward building a large-scale trapped ion quantum computer still remains: the development of a detailed blueprint for the individual modules that need to be capable of performing all required fundamental quantum operations and ideally act as a stand-alone small-scale quantum processor. Each module must also offer efficient connections with additional modules to create a universal quantum computer architecture.

In previously proposed trapped ion quantum computing architectures, modules are powered by laser-driven single- and multiqubit gates. However, the vast amount of individually controlled and stabilized laser beams required in such architectures would make the required engineering to build a large-scale quantum computer challenging. Here, we propose an architecture that is based on a concept involving global long-wavelength radiation and locally applied magnetic fields (*12*). The gate interactions are based on a mechanism first proposed by Mintert and Wunderlich in 2001 (*13*), making use of magnetic field gradients within dedicated gate zones. Only global laser light fields are required for loading, Doppler cooling, and state preparation and readout of ions, whereas laser-driven quantum gates requiring careful alignment in each gate zone are not required in our approach. Large-scale quantum computers, which rely on laser gates and are capable of solving classically intractable problems, may require millions of individual laser beams that have to be precisely aligned with respect to individual entanglement regions and need to be individually controlled. In our microwave-based architecture, all laser fields do not have to be precisely aligned or individually controlled. However, one should note that our architecture still incorporates a number of technical challenges, such as the creation of strong magnetic field gradients and the requirement of calibration operations and well-controlled voltages, which are required to execute quantum gates. We present the blueprint for a scalable microwave trapped ion quantum computer module, which is based on today’s silicon semiconductor and ion trap technology. The modules, driven by global laser and microwave fields, perform ion loading and ion shuttling, generate locally addressable magnetic fields as well as magnetic field gradients to perform single- and multiqubit gates, and feature on-chip photo detectors for state readout. All gate, shuttling, and state readout operations are controlled by on-chip electronics, and a cooling system is integrated into the module to allow for efficient temperature management. Each module, when placed in an ultrahigh vacuum (UHV) system and powered by global laser and microwave fields, operates as a modular stand-alone quantum computer.

Architectures based on photonic interconnects have great potential for scaling up quantum computing (*9*, *10*); however, the interaction rate between modules and therefore the speed of a computer based on these is typically slow (*14*) compared to the execution time of other quantum operations (*2*, *4*, *15*). We propose an alternative method of scaling to a large number of modules based on technology that aligns modules next to each other, enabling ion transport between adjacent modules. A universal two-dimensional architecture is then formed by fast transport (*16*, *17*) of qubits from one module to adjacent modules. A suitable high error–threshold error correction code that only relies on nearest-neighbor interactions was developed by Fowler *et al.* (*18*) and can be implemented using this architecture.

With appropriate modifications, photonic interconnect regions can be added to this blueprint, therefore allowing our microwave trapped ion quantum computer modules to also be connected using photonic interconnects, making the modules useful for alternative architectures proposed so far (*9*, *10*).

## RESULTS

### Microwave-based quantum gates

Single- and multiqubit gates, executed with high fidelity, are essential building blocks of a universal quantum computer. For trapped ion quantum computing, internal states of atomic ions serve as qubits, and the Coulomb interaction between closely spaced ions makes conditional quantum gates with two or more qubits possible (*1*). Precisely aligned laser beams have predominantly been used to couple the dynamics of internal qubit states and motional states and thus implement multiqubit gate operations. This has led to the experimental demonstrations of multiqubit gates with up to 14 ions (*19*) and the demonstration of a two-qubit gate in the fault-tolerant regime (*15*). Nevertheless, there are challenges with the abovementioned implementations when trying to scale them up to a large number of qubits and when trying to increase the gate fidelity further to reduce the overall system size. Technical challenges when operating the large number of laser beams required for a large-scale quantum computer system include intensity and phase fluctuations, frequency drifts of the laser output, micrometer-precise beam alignment, beam-pointing instabilities, and nonperfect beam quality. In addition to the technical challenges, off-resonant coupling to states outside of the qubit subspace when using Raman beams can pose an additional challenge.

A promising solution to the stability and scalability challenges that come with using lasers to implement large-scale multiqubit gate operations was proposed by Mintert and Wunderlich in 2001 (*13*) and makes use of microwave radiation in conjunction with a static magnetic field gradient. Microwave radiation has since been used to perform single-qubit gates with unprecedented fidelity (*2*, *20*), featuring an error per gate as low as 10^−6^ (*2*), and when combined with locally adjustable magnetic fields or magnetic field gradients, individual addressing of closely spaced ions has been demonstrated with cross-talk as low as 10^−5^ (*21*).

Coupling between internal states of trapped ions and their motion, necessary for multiqubit gates, is induced by electromagnetic radiation. Because of the long wavelength (on the order of centimeters), this coupling is vanishingly small for free-running microwaves and is, thus, not useful on its own for multiqubit gate operations. However, when adding a static magnetic field gradient, which exerts a force due to the magnetic moment associated with the qubit states of the trapped ion, multiqubit gates can indeed be implemented (*13*). This magnetic field gradient–induced coupling was first used to implement a two-qubit gate between nearest and non-nearest neighbors by Khromova *et al.* (*22*). Besides using a static magnetic field gradient to implement multiqubit gates, one can also make use of microwave near-field gradients (*23*), which has been demonstrated in the pioneering work of Ospelkaus *et al.* (*24*).

A challenge when using the static magnetic field gradient scheme stems from the requirement of the qubit to be made up of at least one magnetic field–sensitive state. This limits the achievable coherence time and gate fidelities because of uncontrolled magnetic field fluctuations (*22*), and measures have to be taken to shield or compensate these fluctuations. An efficient method of obtaining a qubit that is robust against magnetic field noise is by making use of microwave dressed states (*7*, *25*). These dressed states have been shown to exhibit a coherence time three orders of magnitude longer compared to bare magnetic field–sensitive qubit states and have already been combined with a static magnetic field gradient to cool a single ion to the quantum ground state of motion using long-wavelength radiation (*26*). Using these dressed states that give rise to quantum-engineered clock states, a high-fidelity two-qubit gate has recently been demonstrated (*12*), with the method being capable of producing fault-tolerant quantum gates.

We note that recent work has shown the possibility to cancel the carrier transition during two-qubit gate operations, which is expected to permit much faster gates (*7*, *27*). For the design of a scalable quantum computer module, it is highly advantageous to be able to rely on the matured and commercially developed field of microwave engineering, allowing stable microwave and radio frequency (rf) fields to be generated at comparably low cost and, for a typical user, with a fraction of the complexity of laser systems. Furthermore, microwave radiation can naturally address a large spatial volume, making it very useful when scaling a given operation to many ions. Static magnetic field gradient–induced couplings based on the approach outlined by Weidt *et al.* (*12*) will therefore be used as a basis for two-qubit gate operations within individual modules described here.

### Description of individual quantum computer modules

**Fig. 1:**
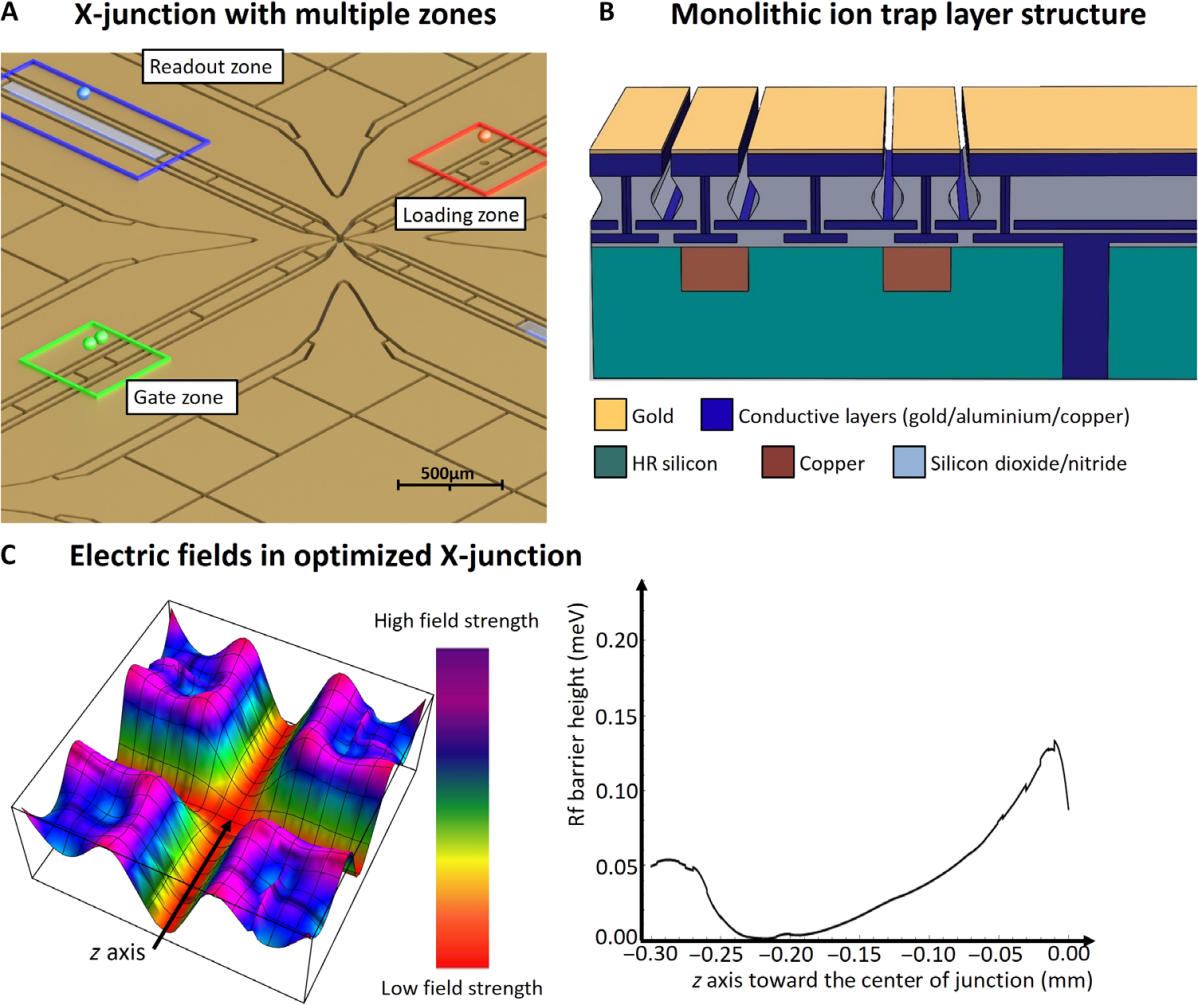
X-junction with multiple zones and corresponding layer structure. (**A**) X-junction featuring multiple zones, including a loading zone (marked red) in selected junction. Multiqubit gates are performed after bringing two or more ions (green balls) together in the gate zone (marked green). The gates are performed by applying a static magnetic field gradient produced by current wires placed underneath the electrodes. State readout is carried out in the readout zone (marked blue) using global laser fields and photodetectors placed underneath the electrodes. (**B**) Layer structure of the ion trap chip consisting of HR silicon substrate and copper current wires embedded in the silicon. Conductive and insulating layers form buried wires, VIAs, through-silicon VIAs (TSVs), and electrodes. (**C**) Left: Illustration of the rf pseudopotential at the ion height of the proposed optimized X-junction geometry. Right: Remaining rf barrier experienced by the ion when moving through the rf minimum along the *z* axis. The rf minimum becomes an rf null inside the entanglement region. This simulation was performed for a ^171^Yb^+^ ion for an ion height of 100 μm and using a drive frequency and voltage of 25 MHz and 200 V, respectively.

We propose a blueprint for a scalable quantum computer module, which makes use of the discussed microwave-based multiqubit gate scheme and is fabricated using silicon microfabrication technology. Each module is a unit cell for a large-scale quantum computer and features microfabricated ion trap X-junction arrays (*28*–*30*). In each X-junction, two or more ions are trapped and feature up to three different zones, as shown in Fig. 1A, including a microwave-based gate zone, a state readout zone, and a loading zone. Once an ion is trapped in the loading zone, high-fidelity ion shuttling operations (*16*, *31*) transfer the ion to the gate zone. There, ions can be individually addressed using locally adjustable magnetic fields and entangled using static magnetic field gradients in conjunction with global microwave and rf fields. When the state of the qubit needs to be detected, the ion is transferred to the readout zone, where global laser fields and on-chip photo detectors are used for state readout. A second ion species is used to sympathetically cool the qubit ion without affecting its internal states (*32*). All coherent quantum operations are performed and controlled by on-chip electronics, relying only on global microwave and rf fields. In our microwave-based architecture, laser light is only required for state preparation and detection, photoionization, and sympathetic cooling. The required laser beams have much less stringent requirements than laser beams for quantum gate realization. The laser beams do not need to have high intensity, and do not need to be phase-stable; the mode profile only requires some overlap with the ion to scatter sufficient photons. Laser beams for sympathetic cooling can even be provided as sheets.

The design of the X-junction, individual zones, control electronics, cooling, and alignment system of the modules will be described in detail in the next paragraphs. We start with the ion trap X-junction and its arms, which constitute the unit cell of the modules and are the core element of the proposed architecture. The gate, detection, and loading zones, which are placed in the arms of the junction, in combination with an optimized design of the junction electrode geometry, which allows for fast high-fidelity ion shuttling and separation, are essential for the operation of the modules. High-fidelity shuttling through junctions requires a highly optimized electrode geometry (*31*, *33*). An example for this junction geometry including its arms is shown in Fig. 1A, featuring minimal rf barrier and barrier gradient shown in Fig. 1C. The optimized electrode geometry is combined with static voltage electrodes designed for fast and efficient ion shuttling and separation (*34*). The rf barrier of the highly optimized X-junction was simulated to be on the order of 0.15 meV for a trap depth of ~80 meV and an ion height of 100 μm. High-fidelity shuttling through X-junctions in a surface trap with a similar barrier has been successfully demonstrated (*31*). We propose the use of fast ion shuttling through the junction, similar to the work presented by Bowler *et al.* (*16*) and Walther *et al.* (*17*), and fast ion separation demonstrated by Bowler *et al.* (*16*) and Ruster *et al.* (*35*) to achieve a quantum computer cycle time on the order of 235 μs.

Decoherence that would be caused by transferring ions through strong magnetic field gradients required for microwave-based quantum gates is avoided by globally turning off the gradient fields during shuttling operations. Remaining spatially varying magnetic fields are compensated for by mapping the magnetic fields in the junctions. Slow variations of the global magnetic field can be detected using dedicated ions at various positions across the module (*36*) and compensated using local magnetic field coils, which will be described in more detail later. Static voltage electrodes are connected using proven and developed vertical interconnect access (VIA) and buried wire technologies (*30*). In addition, a structured ground plane layer is used to avoid exposed dielectrics. The microfabricated conductive and insulating layers are placed on a high-resistivity (HR) silicon substrate exhibiting minimal rf losses (loss tangent, <0.004). The resulting layer structure is shown in Fig. 1B. Using TSV structures, connections to the static and rf electrodes are made from the back of the structure holding the X-junction. The electronic control systems generating the static and rf voltages will be described in more detail in the electronic control section below.

Initial loading of ions and replacement of lost ions are performed using the loading zones placed in one arm of the X-junction close to the edge of each module. Backside loading zones require a global ionization laser beam in combination with an atomic flux originating from the back of the substrate, commonly known as backside loading (*37*). The atomic flux is generated by an atomic oven passing through slots fabricated into the silicon substrate and carefully designed center segmented electrodes, shown in Fig. 1A. The design of these electrodes and the exact shape of the slot were iteratively optimized to obtain an rf potential barrier of the same order or less as an X-junction center. When an ion is lost from a particular position within a module, a new ion is trapped, and all ions placed between the position of the lost ion and the loading zone are shifted by one position, requiring only single-shuttling sequences.

**Fig. 2:**
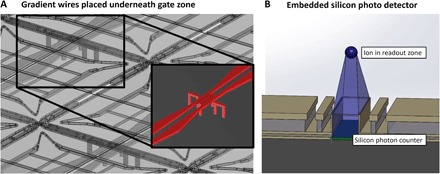
Gradient wires placed underneath each gate zone and embedded silicon photodetector. (**A**) Illustration showing an isometric view of the two main gradient wires placed underneath each gate zone. Short wires are placed locally underneath each gate zone to form coils, which compensate for slowly varying magnetic fields and allow for individual addressing. The wire configuration in each zone can be seen in more detail in the inset. (**B**) Silicon photodetector (marked green) embedded in the silicon substrate, transparent center segmented electrodes, and the possible detection angle are shown. VIA structures are used to prevent optical cross-talk from neighboring readout zones.

A gate zone that features a strong magnetic field gradient and an adjustable local magnetic field offset is located in another arm of the optimized X-junction. The required magnetic field gradients and fields are generated using current-carrying wires and coils embedded in the silicon substrate, as shown in Fig. 2A. Large static magnetic field gradients of 150 T/m at the ions’ position (100 μm above the electrode surface) are used for fast, high-fidelity microwave gates. A current of ~10 A is passed through each copper wire to generate these gradients. Conductivity and cooling of the silicon substrate and copper wires will be discussed in detail in the cooling system description below.

The strong magnetic field gradients work in combination with global long-wavelength radiation fields to perform multiqubit gates in parallel to the entire quantum computer architecture, following the method proposed by Weidt *et al.* (*12*). Here, the correlation between the number of ions and the number of required gate radiation fields vanishes, and only a fixed number of gate radiation fields are needed independent of the number of ions. Therefore, instead of requiring thousands or even millions of individually controllable laser or microwave fields, the method used here only requires a handful of global microwave fields originating from emitters periodically placed within the vacuum chambers to implement the required quantum logic on arbitrarily many ions.

This scaling method can be implemented using two different approaches. The first involves making use of the already present static magnetic field gradient in the gate zone, where the ions can be shuttled along this gradient to change the ions offset magnetic field, utilizing voltages that are applied to the microfabricated ion trap chips, thereby bringing the qubit frequency into resonance with the global microwave fields of choice to perform the desired single- or multiqubit gate. The alternative approach involves using local B-field coils to bring the qubit into resonance. Because the proposed architecture already features local offset coils used to adjust the magnetic field in each gate zone and to compensate slow variations of the local magnetic field, we will focus on the latter approach to shift qubits in and out of resonance with global radiation fields.

The local B-field coils are placed underneath each gate zone and are shown in Fig. 2A. To ensure that they can be used to shift ions in and out of resonance with multiple global microwave fields, we implemented a fast control system. The on-chip control electronics is based on digital-to-analog converters (DACs) that control the currents applied to the very low inductance coils (on the order of 25 nH), with 16-bit precision and an update rate of >1 MHz. Required microwave frequencies for the global fields are generated by commercial frequency generators, and amplifiers supply the signal with sufficient amplitude to emitters inside the system. The gate zones then perform all quantum operations controlled by in-vacuum electronics and powered by global long-wavelength radiation fields.

Ions are precisely placed in the large static magnetic field gradient during the gate operations using highly stable (better than 50 μV) and precise voltages applied to the appropriate voltage electrodes in the gate zones. To perform shuttling operations, these voltages are overridden using the fast control system using a summing amplification circuit.

During shuttling operations, the large static magnetic field gradient could be turned off, removing the requirements on shuttling through large magnetic fields. The wires creating the large static magnetic field gradients will have an impedance on the order of 10 μH, allowing a gradient ramp-up and ramp-down time on the order of 5 μs.

Readout zones are incorporated into the other arm of the X-junction to detect the quantum state of the ions after performing single- and multiqubit gates, as shown in Fig. 1A. In the readout zone, multiple center segmented electrodes are made of indium tin oxide (ITO) instead of gold. ITO is ultraviolet (UV)–transparent (~80% transmission) and allows the light emitted from an ion placed above the zone to pass through the electrodes. Photodetectors are fabricated onto the silicon substrate and separated from the electrodes by a highly UV-transparent dielectric layer, similar to the concept presented in a study by Eltony *et al.* (*38*) and shown in Fig. 2B. VIA wall structures are used to prevent optical cross-talk from neighboring readout zones. Commercial silicon-based microfabricated photon counters (Hamamatsu S12571-100, multi-pixel photon counters) reach quantum efficiencies of ~30% and are compatible with the proposed silicon substrate. When cooled to 77 K, they also show a reduction of dark count rate on the order of 10^5^ to ~1 Hz (*39*).

The total photon detection efficiency of this detection setup will be on the order of 2%, considering an 80% transmission rate of the ITO and dielectric layer and a collection efficiency of ~10%. The detection efficiency and dark count rate are comparable to the values given in the study by Noek *et al.* (*4*), and a similar state readout fidelity on the order of 99.9% for a detection window of 25 μs can therefore be expected for this setup.

State readout operations will have to be performed many times during error-corrected logical qubit operations. To preserve the state of physical qubits performing these operations, only ions placed inside the readout zones are illuminated, whereas ions placed in the gate zones are not. Readout and gate zones are placed in perpendicular arms of junctions, as shown in Fig. 1A. Global laser beams are steered parallel to and between the gate zone arms, which are separated by 2.5 mm, only addressing the ions in the readout zones shown in Fig. 2B. The required accuracy of the beam steering is readily achieved using in-vacuum optics.

The X-junction structures, equipped with the zones discussed, occupy an area of 2.5 × 2.5 mm^2^ and can be fabricated in large numbers on a silicon wafer to form the scalable quantum computer module. A total of 1296 individual X-junctions can be monolithically fabricated onto a 90 × 90–mm^2^ silicon wafer piece, compatible with standard 150-mm wafer sizes. If all of these X-junctions are electrically connected together, the capacitance and power dissipation will become too large to be driven with a standard helical resonator of high-quality factor (*40*).

Simulations performed using the Advanced Design System software tool (Keysight Technologies) show that by connecting 6 × 6 junctions together to form an electrical submodule, the capacitance can be kept below 80 pF, and a quality factor of *Q* > 200 is achievable using a compact helical resonator of ~15 mm in diameter. An additional requirement to achieve a high-quality factor is to use a substrate with low rf loss. Therefore, an HR silicon substrate with a bulk resistivity of 50 kΩ·cm was assumed for these simulations. Compact resonators are placed inside the system underneath the module and connected with shielded cables to the electrical submodules. All resonators are attached to the same frequency source, and the resonant circuits are tuned into resonance with the frequency source using variable capacitors. The close proximity of the electrical sections will lead to capacitive coupling between the resonators and, as a result, lead to phase matching of the resonators and neighboring rf electrodes. Careful design of the connection paths on the ion chips is used to avoid non-negligible phase differences between relevant rf electrodes.

Each electrical submodule features 1224 static voltage electrodes and 108 individual local gradient current wires. The required static voltages and currents are supplied by DACs inside the vacuum system similar to the concept presented by Guise et al. (*41*). DACs are fabricated on separate silicon substrates, which are attached to the ion trap substrate using TSV and wafer-stacking (*42*) technology. Each wafer layer features four DACs with 160 analog outputs in total (the DAC AD5370 has sufficient outputs and was used as an example, but a modified version will be required that operates at higher update rates) and, combined with the required TSV and RC filters, occupies an area of no more than 15 × 15 mm^2^. Generating enough analog outputs requires a total of nine wafer layers that will be stacked together. An additional layer is used to house an electronic control unit, which controls the in-vacuum DACs and detection system.

Each scalable quantum computer module is made up of 6 × 6 electrical submodules, fabricated onto a 90 × 90–mm^2^ HR silicon wafer piece. The module is controlled by on-chip electronics and performs the required quantum operations using magnetic field gradients, local magnetic fields, and global laser and microwave fields. Embedded copper wires generating the magnetic field gradients, shown in Fig. 2A, are routed in such a way that only four high-current connections are required per module.

Passing large currents of 10 A through wires with a small cross section (~30 × 60 μm^2^) makes it essential that the resultant heat is efficiently distributed and transported away from the modules. In addition, the power dissipated by the ion trap structure and the in-vacuum electronics needs to also be transported away from the modules. Melting of the wire structures can be avoided by cooling the silicon substrates to below 100 K, which results in an extremely high thermal conductivity [k >1000 W/(m·K)] of silicon (*43*) and an increase of the copper conductivity by a factor of 10 (*44*). To estimate the temperature of the copper wires in this design, the total heat output per module has to be calculated. Considering the heat generated by the copper wires, rf dissipation in the trap structure, and power dissipation of the on-chip electronics, a maximum heat output per module is estimated to be on the order of 1000 W, which is equal to 0.12 W/mm^2^ and less than that of a modern computer processer unit (Intel Ivy Bridge 4C has a power dissipation of ~0.5 W/mm^2^). A detailed calculation will be given in Materials and Methods. The assumed heat output is likely to be significantly lower because the power dissipation of on-chip electronics and ion trap structures is estimated for room temperature. A liquid nitrogen microchannel cooler is integrated into the back wafer of the modules to efficiently remove the heat from the modules. Deep trenches are etched into the backside of the last wafer, forming channels through which liquid nitrogen is passed. The channels are covered using an additional silicon wafer. Fabricating the entire module including the liquid cooler out of silicon prevents additional stress and wafer bow arising from different thermal expansion coefficients.

A similar microchannel cooler has been shown to achieve a heat transfer coefficient of >0.1 W/(mm^2^·K) (*45*), which is sufficient for this system. On the basis of the total heat dissipation and the thermal conductivity of silicon wafers and copper interconnects, the thermal gradient between the copper wires and the coolant through the multiwafer package and microchannel cooler will only be ~2 K, preventing the copper wires from melting. Liquid nitrogen, which is cooled from 77 to 65 K to prevent boiling inside the microchannel cooler, will be used as a coolant and is supplied to the modules using multiple UHV-compatible flexible steel tubes. Continuous flow liquid nitrogen coolers are commonly used for detectors and, if designed correctly, introduce minimal vibrations to the system (much less than 100-nm amplitude) (*46*).

Each of these modules can work as a stand-alone small-scale quantum processer module featuring 1296 X-junctions. If one wants to perform a computationally hard problem, such as Shor factorizing a 2048-bit number, a much larger architecture consisting of many modules will be required. Each module will have to be interfaced with each other to create a universal quantum computer architecture. The approach presented by Monroe *et al.* (*9*) makes use of photonic interconnects and commercial fibers to interface arbitrarily many modules. Fiber switches can then be used to connect any module with any other module in the architecture. This approach has great potential, but the performance of this system is currently limited by the interaction rate between modules (*14*), which is typically much slower than other quantum operations (*2*, *4*, *15*) performed by the modules.

### Scaling modules to a universal quantum computer architecture

We propose an alternative scheme that does not rely on photonic interconnects and is therefore not limited by their interaction rate. In our approach, modules are designed in such a way that ions can be directly shuttled from one module to another. Rf and static voltage electrodes thus need to be fabricated all the way to the edge of the modules so that the electric fields confining the ions reach beyond the edges. The modules must also be accurately aligned so that two neighboring modules create an overlapping electric field. If such an electric field can be created, ions can be shuttled from one module to another.

The resulting two-dimensional module array will feature fast interaction rates between nearest-neighbor modules, without the need of a special photonic interconnect system. The challenging part of this scheme is to accurately align all modules to each other to prevent large barriers or interruptions of the overlapping electric fields from occurring.

We have performed boundary element method electric field simulations of three-dimensional trap structures to investigate the feasibility of shuttling ions from one module to another, taking into account the possible misalignments between adjacent modules. We have analyzed the electric potential and rf barrier caused by rf rails misaligned in different directions and magnitudes. Results of the simulations show that an rf barrier occurs, similar to that found in the center of an X-junction.

**Fig. 3:**
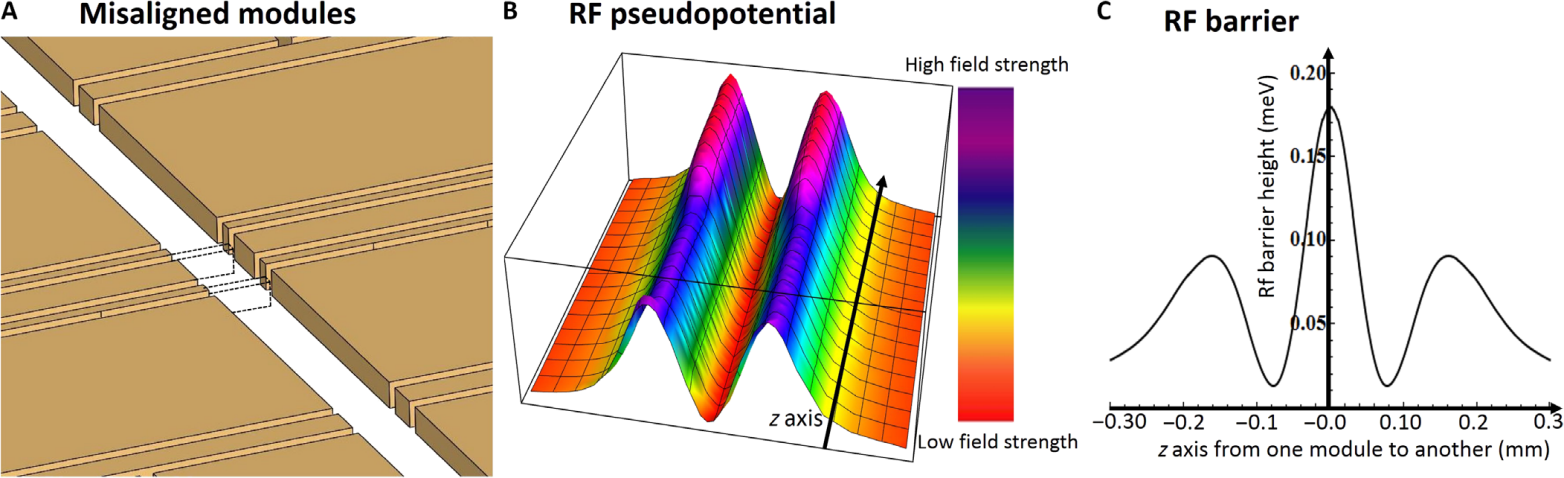
Misaligned modules. Illustration of two modules misaligned in the *xyz* axes by 10 μm each (**A**) and the corresponding rf pseudopotential at the ion height (**B**). (**C**) The resulting rf barrier when moving along the rf minimum in the *z* direction is given in millielectron volt . This simulation was performed for a ^171^Yb^+^ ion for an ion height of 100 μm and using a drive frequency and voltage of 25 MHz and 200 V, respectively.

In the case of a misalignment in all three axes by ≤10 μm, the simulated rf barrier was found to be ≤0.2 meV, as shown in Fig. 3, for a trap depth of ~100 meV and an ion height of 100 μm. The barrier of 0.2 meV is of similar height to the one found in our optimized X-junction center and the one presented in a study by Wright *et al.* (*31*), where high-fidelity shuttling was successfully demonstrated. Shuttling fidelities comparable or higher than those through the X-junction centers can therefore be expected if the rf voltages applied to both modules show no significant phase or frequency difference, and the neighboring modules can be aligned with an accuracy of ≤10 μm.

As discussed in the previous section, all resonators providing the rf voltages to the modules are connected to the same frequency source. In addition, all modules feature the same path length and impedance of the coaxial connections from the resonators to the rf electrodes. This will result in a negligible rf phase difference of the electric fields generated by adjacent modules, which could otherwise weaken the trap depth at the intersection between modules.

**Fig. 4:**
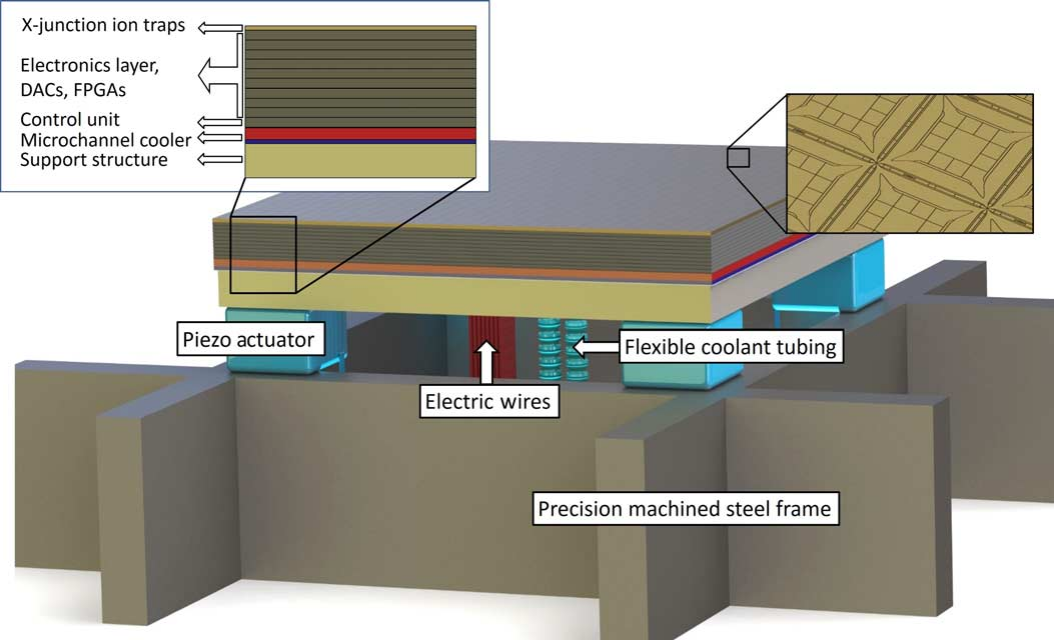
Scalable module illustration. One module consisting of 36 × 36 junctions placed on the supporting steel frame structure: Nine wafers containing the required DACs and control electronics are placed between the wafer holding 36 × 36 junctions and the microchannel cooler (red layer) providing the cooling. X-Y-Z piezo actuators are placed in the four corners on top of the steel frame, allowing for accurate alignment of the module. Flexible electric wires supply voltages, currents, and control signals to the DACs and control electronics, such as field-programmable gate arrays (FPGAs). Coolant is supplied to the microchannel cooler layer via two flexible steel tubes placed in the center of the modules.

Precision machined steel frames are mounted inside the vacuum chambers to achieve an alignment of the modules with an accuracy of ≤10 μm in three dimensions. The planarized top surface of the steel frames is characterized using an interferometric measurement system, and the modules are mounted on the surface using a high-precision die bonder tool. To increase the alignment accuracy and to allow for drift compensation, multiple UHV- and cryogenic-compatible XYZ piezo actuators are placed between the bottom of each module and the top of the steel frames. The illustration in Fig. 4 shows a pictorial representation of a single module of the scalable architecture including required connections and attached piezos on the top of a steel frame.

The exact positions of the modules are determined using microfabricated diffraction gratings on the front of the modules in combination with a laser measurement system. The alignment system determines the position of neighboring modules and corrects for misalignment using the piezo actuators and, if required, the die bonder tool.

The requirements for the high-precision position measurement system and the module placement are technically very challenging but, on a smaller scale, have been implemented with much higher precision using lithography stepper systems where 3- to 5-nm alignment precision of large-wafer stages in vacuum is routinely achieved (ASML TWINSCAN NXE:3300B).

The discussed alignment capability would be severely hindered if the modules are strongly warped in random directions or the edges of the modules are not precisely fabricated. Because of the large thickness of the silicon wafer modules (on the order of 10 mm), negligible wafer bow is expected, and the bow will also be characterized for all modules before assembly. The edges of the modules are created using high-resolution photolithography and anisotropic dry etching (Bosch process). These process steps, which are commonly used in the microfabrication of microchips and microelectromechanical system devices, reach submicrometer precision and will not limit the alignment capabilities. In addition, only the top wafer carrying the ion trap structures will feature the full footprint size of the module, and all other wafers, containing control and detection electronics and cooler, will have a slightly smaller footprint.

**Fig. 5:**
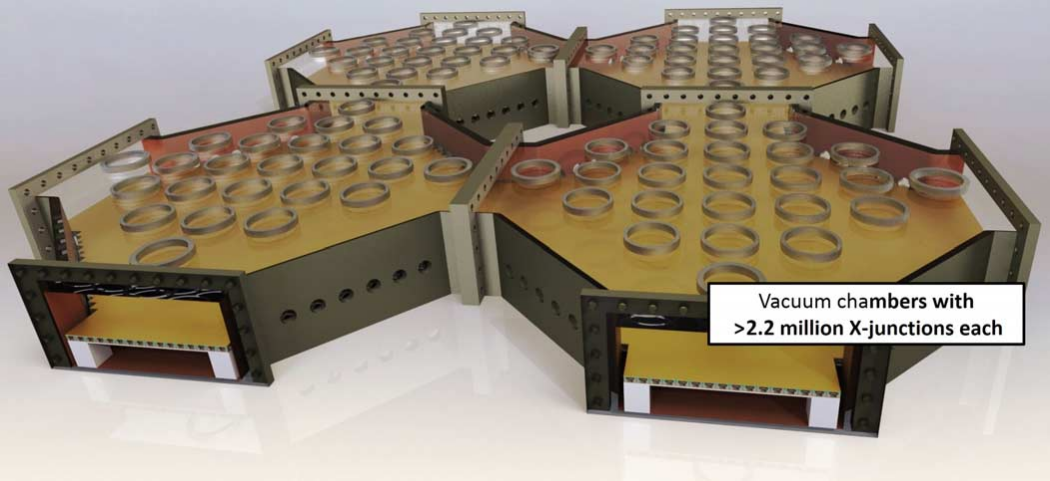
Illustration of vacuum chambers. Schematic of octagonal UHV chambers connected together; each chamber is 4.5 × 4.5 m^2^ large and can hold >2.2 million individual X-junctions placed on steel frames.

The discussed steel frames are accurately placed inside large octagon-shaped UHV chambers (~4.5 × 4.5 m^2^ large), which feature viewports on the sides and top to allow for optical access, as shown in Fig. 5. Imaging, beam shaping, and guiding the laser light fields above the trap surfaces are achieved using in-vacuum optics. Each chamber also incorporates all required feedthroughs for currents, static voltages, rf and microwave signals, coolant, and digital control signals. In addition, the chambers are equipped with liquid nitrogen–cooled heat shields and a variety of vacuum pumps to create an UHV environment. Multiple chambers can be directly bolted and welded together with a monolithic steel frame passing through all the vacuum chambers, creating a modular universal quantum computer of large size. Each chamber shown in Fig. 5 can hold ≥2.2 million individual junctions. Although the technical feasibility of our approach has been demonstrated above, it should be noted that the required engineering is certainly not simple.

### Surface error correction code

Performing computationally hard problems using a quantum computer, such as prime factoring of a 2048-bit number, requires logical operations to be performed with a much lower error rate than achievable by any potential quantum system and will prohibit a successful outcome of such a computation. Quantum error correction, which uses multiple physical qubits to create logical qubits with a much lower error rate, is thus a necessity for scalable quantum computing. Following the pioneering work by Steane (*47*) and Shor (*48*), a variety of different error correction strategies have been developed. In the original proposals, logical qubits were encoded in a number of physical qubits using special code word states that allow identification and fixing of errors (occurring on single physical qubits) without destroying the logical qubit states. These codes require the error probabilities associated with each operation on the physical qubits to be below a very challenging threshold for the error code to work and even lower error rates [on the order of 10^−5^ (*47*)] for a practical implementation with manageable resource requirements.

Since then, schemes have been developed that can tolerate much larger error probabilities but rely on the coding of logical qubits in a larger number of physical qubits compared to the previously discussed error correction codes. One such scheme is the surface error correction code described by Fowler *et al.* (*49*), which tolerates gate error probabilities of up to 10^−2^ and relies only on nearest-neighbor interactions. We will briefly summarize how the implementation of the surface code protects from errors, and discuss how it can be implemented with this two-dimensional array architecture and can be used to perform fault-tolerant logic operations.

**Fig. 6:**
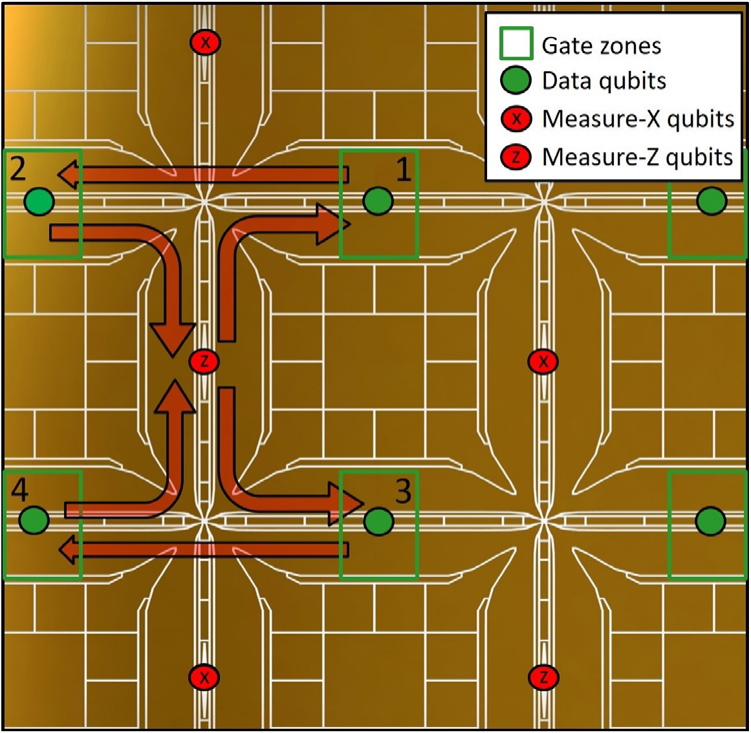
Error correction code sequence. Small section of the scalable architecture illustrating how data and measurement qubits interact with each other in the gate zones to execute the surface code. Data qubits are static, and measurement qubits are shuttled to all adjacent gate zones.

The surface code requires physical qubits to be placed in a regular lattice, which we can decompose into one sublattice holding so-called data qubits and another sublattice holding measure-X and measure-Z qubits. In our architecture, two ions are trapped in each X-junction section, as shown in Fig. 6. One ion is permanently placed in the gate zone, constituting the data qubit, and the second ion is alternatively a measure-X or measure-Z qubit, placed in the readout zone and can be shuttled to the four adjacent data qubits.

Measure-X and measure-Z qubits constantly monitor the states of their four nearest-neighbor data qubits. The measure-Z qubits perform four successive controlled NOT (CNOT) gates, with the data qubits in their respective gate zones in the order displayed in Fig. 6, after which the state of the measure-Z qubit is detected. The measure-X qubits perform almost the same sequence, but an additional Hadamard gate is applied to them before and after the four CNOT gates. The measure qubit sequence is run simultaneously in a synchronized manner with all measure qubits of the entire architecture and repeats itself over and over again throughout the calculation. These sequences of four CNOT gates (two additional Hadamard gates for Z-errors) are designed to perform parity checks of the fourfold σ_*x*_ and σ_*z*_ operators that allow for error detection on the surface code. The eigenstates of the products are dependent on the four neighboring data qubits, and all products commute with one another. If a single data qubit of the array undergoes an error, it will result in eigenvalue flips of the two adjacent operators, and the error is identified by the surface code at the end of the error chain. The error could then be corrected, but an easier and more robust way is to merely store the error information. The correction can then be performed “in software,” that is, by translating the measurement results at the end of execution into the appropriately modified values.

Should ions be lost from the qubit space due to entering a dark state, optical pumping is used to drive them back to the qubit space, and the net effect is then equivalent to a qubit error of the form detected and corrected by the code. Complete loss of a measure qubit due to collisions with background gas is detected due to the periodic state readout of these after each measurement cycle. When this loss is detected, the empty measure qubit site is replenished with a new ion from one of the loading zones by shifting all ions between the loading zones and the empty measure site. The loss of a data qubit cannot be detected directly; we therefore propose to use the measure qubit as an indicator of whether a data qubit is present in the gate zone.

When the measure qubit is shuttled into the gate zone, it will experience a different electric potential if a data qubit is present. If the data qubit is present, the two ions occupy left and right potential minima, whereas if the data qubit ion is missing, the measure qubit will occupy the center of the potential well. When shuttling the measure qubit out of the gate zone, a special potential well is applied that leaves measure qubits placed in the center of the well, whereas off-center measure qubits are extracted and the measurement cycle proceeds. The measure qubit automatically replaces missing data qubits and is replenished in a later cycle using the previously discussed method. Typically, after a single execution of the measurement cycle, the surface code cycle is reestablished.

In an arbitrarily sized two-dimensional array of qubits protected by the surface code, only a single unique quantum state exists. To implement logical qubits in this protected array, additional degrees of freedom need to be added by turning off the readout carried out by two measure-X or measure-Z qubits at a distance of a few sites (*49*).

Logical one- and two-qubit gates are implemented by so-called braiding operations, which are described in more detail in the study by Fowler *et al.* (*49*). Braiding operations can be implemented in our surface code–protected architecture by “switching” on and off multiple measure qubits in such a way that they form paths (braids) that lead from the measure qubits (defining one logical qubit), around the measure qubits (defining another logical qubit), and back. After a complete cycle where the measure qubits are switched on again along the same path, a logical two-qubit gate is effectively implemented on the logical qubits. While performing this logical qubit gate, the physical qubits in the array do not have to perform any additional operations besides the ones required for the error correction cycle. Only the regions of the lattice forming the defects have to be switched off, corresponding to data qubits remaining in their gate zone and measure qubits being “parked” in their readout zone. The defect regions are not constant in space/time for the entire logic operations, and therefore, the regions will be switched on and off at different times, depending on the topological geometry of the circuit. When a region is switched back on, the error correction cycle is resumed, and the physical qubits perform their designated operations again.

Non-Clifford single-qubit π/8 Z-rotations are the most challenging operations to implement with the surface code. In Fowler *et al.*’s study (*49*), it is proposed to implement these single-qubit gates by performing logical two-qubit CNOT gates using ancilla qubits, which are logical qubits initialized in states of the form |0〉_*L*_ + *e*^*i*π/4^|1〉_*L*_. Logical qubits cannot be directly initialized to such a state, performing only logical gate operations. It was therefore proposed (*49*) to perform the required rotations on the physical state of one data qubit. The data qubit state is then “injected” into an error-corrected logical qubit. Simplistically, a logical qubit consisting of only one data qubit is created, and the rotation is performed on the data qubit state, defining the logical qubit state. Afterward, the logical qubit is “grown” to achieve the desired fault tolerance again. Although the initialized state is now error-protected, the original operation creating the state was performed on a physical qubit and does not have a low enough error probability for performing computationally hard problems. Therefore, it is necessary to create multiple logical qubits with the same injected states and to use an error correction code like the Steane code to distill them to high fidelity. The Steane code is implemented using the surface code logical qubit operations and produces logical ancilla qubits, which are needed in large numbers. Although their production with adequate precision is the most challenging operation [it is estimated to occupy more than 90% of all physical qubits of the system (*49*)], the physical qubits do not have to perform any additional or more complex operations compared to the standard error correction cycle.

## DISCUSSION

We define modularity in the first instance as a fabrication modularity, meaning, individual modules are fabricated and may be exchanged if faulty. This is important for the engineering of the system, in particular when imagining large-scale systems. When using the surface code, the basic physical hardware is typically a uniform two-dimensional array with nearest-neighbor gates only. Clustered interactions that may exhibit modulation in the connectivity graph could exist at the logical level, and the surface code supports the interaction of arbitrary pairs of logical qubits. By implementing and amending the surface code scheme as outlined above to accommodate one measure and one data qubit ion in each X-junction of our architecture, we have the necessary ingredients to use the scheme analyzed in detail in Fowler *et al*.’s study (*49*). We assume the same gate and memory error probability of 0.1%, which can be achieved when implementing the microwave gate scheme (*12*) with the proposed magnetic field gradient. On the basis of the same scheme, we can give quantitative estimates on the system size and processing time for a machine that solves a relevant, hard problem, such as the Shor factoring of a 2048-bit number. For the calculations, we assume a single-qubit gate time of 2.5 μs, two-qubit gate time of 10 μs, ion separation and shuttling time of 15 μs each, static magnetic field gradient ramp-up and ramp-down time of 5 μs each, and a measurement time of 25 μs, resulting in a total error correction cycle time of 235 μs. On the basis of these numbers, performing a 2048-bit number Shor factorization will take on the order of 110 days and require a system size of 2 × 10^9^ trapped ions. Shor factoring of a 1024-bit number will take on the order of 14 days. Both of these factorizations will require almost the same amount of physical qubits because the required pace of the ancilla qubit generation is the same for a 2048-bit and a 1024-bit factorization. Trapping 2 × 10^9^ ions will require 23 × 23 vacuum chambers occupying an area of ca. 103.5 × 103.5 m^2^.

We believe that these numbers are very encouraging, and we are confident that further improvements to the error correction code could bring down the overhead of performing this calculation by up to an order of magnitude. Assuming that it will also be possible to reduce the error rate of each quantum operation below 0.01%, it would be possible to perform the 2048-bit number factorization in approximately 10 days, requiring on the order of 5 × 10^8^ ions. In addition, one could implement medium-range shuttling, of approximately 30 junctions, which, despite an increase in cycle time, could lead to a further reduction of the required number of ions to 1.1 × 10^7^ ions or 5.5 × 10^6^ X-junctions. These improvements could be implemented without major changes to the modules or the architecture, and all required qubits would fit into three vacuum chambers.

The presented blueprint for a scalable trapped ion quantum computer module combines the advantages of microwave-based quantum gates with on-chip control electronics, which not only generate voltages to perform shuttling operations but also control all quantum information operations. When placed in a vacuum system, which supplies the modules with static and rf voltages, coolant, and global laser and long-wavelength radiation fields, each module can work as a small stand-alone quantum computer. Local adjustable magnetic fields in combination with a small number of global long-wavelength radiation fields can be used for selective addressing of arbitrarily many qubits in parallel (*12*). This has the critical advantage of replacing thousands or millions of laser beams required in previously proposed architectures (*9*, *10*), with only a few global microwave fields, making this technology an efficient engineering solution to construct a large-scale universal quantum computer.

To go beyond a single module and scale to an arbitrarily large quantum computer, we propose an alignment system, making it possible to accurately align modules with their adjacent ones. The resulting system features nearest-neighbor ion-ion interactions spanning across the entire architecture, making it suitable for the surface error correction code. By adding photonic interconnect regions, the modules can also be used in alternative scalable quantum computing architectures.

To demonstrate some of the core aspects of this architecture, one could carry out proof-of-principle experiments, such as the alignment of two microfabricated ion trap chips with each other as well as demonstrating ion transport between these modules. The next steps would then include combining these transport operations with coherent single- and two-qubit gate operations toward realizing a logical qubit.

We have described an engineering blueprint for a microwave-based trapped ion quantum computer based on modules that are connected via ion shuttling, forming a scalable quantum computer architecture. The proposed modules combine the advantages of microwave-based quantum gates with local offset B-fields to remove the correlation between the number of qubits in the quantum computer and the required number of radiation fields to perform the quantum logic operations. We have shown how the surface error correction code can be implemented in this architecture and have given an estimate of the system size and execution time required to perform a 2048- and 1024-bit number Shor factorization in this scalable architecture.

## MATERIALS AND METHODS

The electric potential experienced by the ion when shuttled through junctions and between physically separate modules (Fig. 3) was simulated using the “Essential Numerical Tools Packages” by K. Singer (*50*) and using Wolfram Mathematica. Calculations for system sizes and computation speeds using the error correction code were based on the work presented by Fowler *et al.* (*49*).

Calculations for power dissipation and thermal gradients of the scalable modules were performed, assuming a current of 10 A is passed through the narrowest wire section of 30 × 60 μm^2^. The wires are made of copper, for which the electrical resistivity is assumed to be 2 nΩ·m (*44*) and the thermal conductivity to be κ ~ 482 W/(m·K) at 100 K. The copper wires are embedded in a silicon substrate with a thermal conductivity of κ ~ 1000 W/(m·K) at 100 K (*43*) using a thin (50 nm) titanium layer for adhesion with a thermal conductivity of κ ~ 31 W/(m·K) at 100 K. Assuming the heat spreads inside the 10-mm-thick silicon stacked wafers at an angle of at least 36.5° (*51*), the temperature difference between the copper wires and the microchannel made of silicon placed at the bottom of the stacked wafers was calculated to be less than 1 K.

Before one can calculate the total temperature difference between the copper wires and coolant, we need to determine the total power dissipation that has to be cooled by the microchannel cooler. To calculate the power dissipated by the currents passing through one X-junction, we assumed that there are two copper wires with an average width of 125 μm, a height of 30 μm, and a length of 2.5 mm. The resulting total power dissipation contribution from wires at 100 K is ~0.3 W for one X-junction section and ~350 W per module. The power dissipation of the DACs and electronics was estimated on the basis of room temperature values given, for example, ~0.3 W for DAC AD5370, and resulted in an additional ~350 W of power dissipation. Control electronics and rf power dissipation of each module were estimated to be ~300 W in total, based on power dissipation values for field-programmable gate arrays and rf simulations. The total power dissipation will therefore be on the order of 1000 W, and the power density per module will be ~0.12 W/mm^2^, which is significantly lower than a modern central processing unit (Intel Ivy Bridge 4C has a power dissipation of ~0.5 W/mm^2^). All of these estimates are based on room temperature values and are expected to be significantly lower when operated at <100 K.

Assuming that the built-in microchannel cooler achieves a comparable heat transfer coefficient between the cooler and the coolant to the one published in the study by Riddle and Bernhardt (*45*), for example, >0.1 W/(mm^2^·K), the total temperature difference will be lower than 2 K. On the basis of the anticipated temperature of the coolant of <70 K, the resulting surface temperature of the module is expected to be ~72 K.

## Supplementary Material

http://advances.sciencemag.org/cgi/content/full/3/2/e1601540/DC1

## SUPPLEMENTARY MATERIALS

Supplementary material for this article is available at http://advances.sciencemag.org/cgi/content/full/3/2/e1601540/DC1 movie S1. Animation illustrating the system design and operation of a microwave trapped ion quantum computer.
