# Supplementary material for: Blueprint for a microwave trapped ion quantum computer
Source: Sci Adv. 2017 Feb 1;3(2):e1601540. doi: 10.1126/sciadv.1601540 (PMC5287699; doi:10.1126/sciadv.1601540)
Supplement: http://advances.sciencemag.org/cgi/content/full/3/2/e1601540/DC1 [file supp_3_2_e1601540__index.html]

Science Advances | Science Advances

## Supplementary Materials

**Other Supplementary Material for this manuscript includes the following:**

- movie S1 (.mp4 format). Animation illustrating the system design and operation of a microwave trapped ion quantum computer.

**Files in this Data Supplement:**

- Adobe PDF - 1601540\_SM.pdf
