# Supplementary material for: Blueprint for a microwave trapped ion quantum computer
Source: Sci Adv. 2017 Feb 1;3(2):e1601540. doi: 10.1126/sciadv.1601540 (PMC5287699; doi:10.1126/sciadv.1601540)
Supplement: http://advances.sciencemag.org/cgi/content/full/3/2/e1601540/DC1 [file 1601540_SM.pdf]

Science Advances

AAAS

advances.sciencemag.org/cgi/content/full/3/2/e1601540/DC1

Supplementary Materials for

Blueprint for a microwave trapped ion quantum computer

Bjoern Lekitsch, Sebastian Weidt, Austin G. Fowler, Klaus Mølmer, Simon J. Devitt, Christof Wunderlich, Winfried K. Hensinger

Published 1 February 2017, *Sci. Adv.* **3**, e1601540 (2017)
DOI: 10.1126/sciadv.1601540

Other Supplementary Material for this manuscript includes the following:
(available at advances.sciencemag.org/cgi/content/full/3/2/e1601540/DC1)

- movie S1 (.mp4 format). Animation illustrating the system design and operation of a microwave trapped ion quantum computer.
